# Effects of pollination limitation and seed predation on female reproductive success of a deceptive orchid

**DOI:** 10.1093/aobpla/plu031

**Published:** 2014-06-09

**Authors:** Ryan P. Walsh, Paige M. Arnold, Helen J. Michaels

**Affiliations:** Department of Biological Sciences, Bowling Green State University, Bowling Green, OH 43402, USA

**Keywords:** Conservation, orchid, plant reproduction, plant–insect interactions, pollen limitation, pollination ecology, reproductive trade-offs, seed predation, supplemental pollination.

## Abstract

For many species of conservation significance, multiple factors limit reproduction. This research examines the contributions of plant height, number of flowers, number of stems, pollen limitation and seed predation to female reproductive success in the deceit-pollinated orchid, *Cypripedium candidum*. The deceptive pollination strategy employed by many orchids often results in high levels of pollen limitation. While increased floral display size may attract pollinators, *C. candidum*'s multiple, synchronously flowering stems could promote selfing and also increase attack by weevil seed predators. To understand the joint impacts of mutualists and antagonists, we examined pollen limitation, seed predation and the effects of pollen source over two flowering seasons (2009 and 2011) in Ohio. In 2009, 36 pairs of plants size-matched by flower number, receiving either supplemental hand or open pollination, were scored for fruit maturation, mass of seeds and seed predation. Pollen supplementation increased proportion of flowers maturing into fruit, with 87 % fruit set when hand pollinated compared with 46 % for naturally pollinated flowers. Inflorescence height had a strong effect, as taller inflorescences had higher initial fruit set, while shorter stems had higher predation. Seed predation was seen in 73 % of all fruits. A parallel 2011 experiment that included a self-pollination treatment and excluded seed predators found initial and final fruit set were higher in the self and outcross pollination treatments than in the open-pollinated treatment. However, seed mass was higher in both open pollinated and outcross pollination treatments compared with hand self-pollinated. We found greater female reproductive success for taller flowering stems that simultaneously benefited from increased pollination and reduced seed predation. These studies suggest that this species is under strong reinforcing selection to increase allocation to flowering stem height. Our results may help explain the factors limiting seed production in other *Cypripedium* and further emphasize the importance of management in orchid conservation.

## Abstract (precis)

This research examines the contribution of plant height, number of flowers, number of stems, as well the joint impacts of mutualists and antagonists on the pollination biology and seed production of the imperiled, deceptive orchid, *Cypripedium candidum*. We found flowering stem height to be the only morphological feature significant in reproduction, with taller flowering stems simultaneously receiving increased pollination and decreased seed predation. Furthermore we found decreased seed mass in individuals subjected to hand-self pollination treatments. Our results may help explain the factors limiting seed production in other *Cypripedium* and further emphasize the importance of management in orchid conservation.

## Introduction

The complex dynamics between plants, pollinators and seed predators, and how these interactions affect plant reproduction, are important in understanding the evolution of plant floral displays (Irwin and Brody 2011). While many have examined the roles of both mutualists and antagonists in plant reproduction (Gómez 2003; Strauss and Irwin 2004; Abdala-Roberts *et al*. 2009; Burkhardt *et al*. 2009; Carlson and Holsinger 2010; Kolb and Ehrlén 2010; Ågren *et al*. 2013), relatively few have examined the effect of these interactions on the selection of floral traits (Cariveau *et al*. 2004; Parachnowitsch and Caruso 2008). Both mutualists and antagonists may forage on individuals based on floral display size, where larger floral displays provide concentrated resources for pollinators (Peakall 1989; Brody and Mitchell 1997; Mitchell *et al*. 2004) as well as seed predators (Stephens and Myers 2012). The goal of this study was to assess the relationships among mutualists, antagonists and floral display size, and how these biotic interactions influence reproductive success in a deceptive pollination system of conservation concern.

Maximizing pollen transfer efficiency has greatly shaped the evolution of a multitude of floral forms and functions in angiosperms (Barrett 2003). Increased floral display size is expected to increase pollinator attraction and visitation (Peakall 1989; Burd 1995; Aragón and Ackerman 2004; Grindeland *et al*. 2005; Li *et al*. 2011; Sletvold and Ågren 2011). In an experimental manipulation of floral display size in *Mimulus ringens*, Mitchell *et al*. (2004) found that bumblebee pollinators strongly respond to floral display size, probing more flowers in sequence on plants with large numbers of flowers. Larger floral displays also increase plant visibility, thereby increasing the attraction of pollinators from greater distances (Kindlmann and Jersáková 2006). Flowering stem height may similarly increase visibility and pollination efficiency. Synchronous flowering within a plant may also effectively increase floral display size, potentially increasing individual visibility, while synchronous flowering on the population level may increase conspecific competition for the limited pollinator resources (Crone and Lesica 2004; Crone *et al*. 2009). Measures of plant size such as numbers of stems and leaves, which may or may not correlate with floral display size, while not directly affecting the recruitment of pollinators, represent increased photosynthetic resources available for subsequent fruit maturation (Bazzaz *et al*. 1987).

Many orchids rely on a deceptive pollination strategy, a pollination strategy in which the flower provides floral cues indicating a food reward while not providing that reward (Faegri and van der Pijl 1971; Cozzolino and Widmer 2005). Deceptive pollination systems often show lower visitation and pollination relative to rewarding relatives (Nilsson 1980, 1984). To account for this reduced visitation and pollination, some have hypothesized that deception reduces geitonogamous pollination by causing pollinators to flee non-rewarding patches (Smithson 2002; Johnson *et al*. 2004; Schiestl 2005; Kindlmann and Jersáková 2006; Sun *et al.* 2009), referred to as the outcrossing hypothesis (Jersáková *et al*. 2006). Orchids relying on food deception often depend on newly emergent or otherwise inexperienced insects for pollinator services (Jersáková *et al*. 2006). In *Dactylorhiza lapponica*, Sletvold *et al*. (2010) demonstrated strong pollinator-mediated selection on spur length and plant height in the open lawn community of a Norwegian fen. This and other studies of deceptive species (O'Connell and Johnston 1998; Johnson and Nilsson 1999; Gigord *et al*. 2001) indicate that a variety of floral traits (plant height, flower number, petal colour) may be targets of selection for increasing female reproductive success through pollinator visitation.

Pollen limitation, defined as the difference in seed production between open pollinated (natural pollination with no supplementation) and supplemental pollination treatments, occurs when the average open-pollinated seed production is significantly less than the average seed production of individuals receiving supplemental pollen (Knight 2003). While increased floral display size may reduce pollinator limitation, it may also substantially increase geitonogamy (de Jong *et al*. 1993; Barrett and Harder 1996; Snow *et al*. 1996; Galloway *et al*. 2002). Geitonogamy reduces female function by reducing the number or quality of the offspring, but also impacts male function by reducing the quantity of pollen available for export to other plants, also known as pollen discounting (Harder and Barrett 1995; Barrett 2003; Johnson *et al*. 2004).

After pollination, female reproductive success may be eroded by seed predation. Pre-dispersal seed predation can play an important role in determining fecundity and long-term population persistence (Louda and Potvin 1995; Russell *et al*. 2010). Chronic seed predation rates can limit population growth by reducing fecundity, while stochastic predation rates can play a more diffuse, but equally important role in population dynamics (Kolb *et al*. 2007). Just as pollinators are often attracted to large floral displays, seed predators may be attracted to the accompanying large ovule resource (Brody and Mitchell 1997; Kudoh and Whigham 1998; Galen and Cuba 2001; Irwin *et al.* 2003; Adler and Bronstein 2004; Shimamura *et al*. 2005; Stephens and Myers 2012) as many seed predators rely on ovule development to feed their offspring (Cariveau *et al*. 2004). Therefore, although dense floral resources may attract mutualist pollinators, the accompanying dense floral and fruit resources may simultaneously attract antagonist herbivores and seed predators, creating conflicting selective pressures (Louda and Potvin 1995; Strauss and Irwin 2004; Ågren *et al*. 2008). Similar to the influence of co-flowering species on plant–pollinator interactions, rates of pre-dispersal seed predation also can be influenced by community context through their attraction to other species that share seed predators (Recart *et al*. 2013).

*Cypripedium* species are deceptive, deciduous, terrestrial orchids with growth emerging from a subterranean rhizome (Stoutamire 1967). *Cypripedium candidum* Muhlenberg ex. Willdenow, the Small White Lady's Slipper, with yellow-green lateral sepals and petals with a white, purple-spotted labellum (Stoutamire 1967), occurs in calcareous prairies as well as fens and limestone barrens (Cusick 1980). The plants occur as single plants or large clumps (1–12 vegetative stems) containing a single large flower per vegetative stem. This floral architecture, combined with a short flowering period, severely limits the probability of reproduction by restricting opportunities for pollination. *Cypripedium candidum* flowers are pollinated by small (4–6 mm long) adrenid and halictid bees (Catling and Knerer 1980; Bowles 1983; Wake 2007). While pollen transport distances have not been specifically studied for adrenid and halictid bees, previous studies have found pollinia transport distances to vary widely (Nilsson *et al*. 1992; Kropf and Renner 2008). In the larger (8–17 mm long) *Andrena* bee species, Kropf and Renner (2008) found a maximum transport distance of 6.9 m. Data collected in a separate study (Walsh 2013) in Ohio *C. candidum* found pollen dispersal to be limited to 1 m within the focal plant.

The primary antagonist for *C. candidum*, *Stethobaris ovata* (Family: Curculiondae; subfamily: Baridinae), is a known weevil seed predator of *Cypripedium* spp. and other temperate orchid genera, with reports of adults in Canadian populations feeding on emerging shoots and flower buds (Light and MacConaill 2011). Adult weevils emerge in early spring along with *Cypripedium* shoots, and oviposit in developing fruits and, possibly stems, resulting in either fruit abortion or near total loss of the developing embryos (Light and MacConaill 2011). Predation rates on *C. parviflorum* in Canada vary from 32 to 53 % among plants in a population, depending on climate and availability of fruit resources (Light and MacConaill 2002). Little is known about the life history of the weevil, although they may complete two life cycles within a growing season (M. Light, pers. comm.).

The goal of this study was to assess the effects of plant and floral display sizes on both pollination and seed predation, and understand how these factors influence female reproductive success in the long-lived, highly specialized deceptive orchid, *C. candidum. Cypripedium candidum* is highly dependent on full sun in open areas and, as with many prairie species, populations begin to decline with the invasion of woody plants (Curtis 1946). In addition to the potential shading effects of encroaching woody vegetation, increased heterospecific stem density has been shown to reduce pollination and population recruitment (Wake 2007). Although the major challenges in orchid conservation research reside in understanding their symbiotic associations with fungi and improving survival following propagation (Krupnick *et al*. 2013), effective management and restoration will require a more mechanistic understanding of how habitat changes influence all biotic interactions limiting recruitment. We hypothesized that an increased floral display size would (i) increase pollinator visitation as indicated by increasing fruit set, (ii) increase geitonogamy, resulting in increased fruit abortion, decreasing fruit maturation and offspring fitness, and (iii) increase attraction of antagonists by providing an attractive resource concentration for seed predators. To address these hypotheses, we conducted two pollen limitation experiments over the course of the 2009 and 2011 field seasons in two separate sites, examining the effects of plant size on pollinator limitation and seed predation in 2009, and the effect of pollen quality in a seed predator exclusion experiment in 2011.

## Methods

The primary field site for this study was located in Northern Ohio (GPS coordinates available upon request). Historically *C. candidum* existed in at least seven Ohio counties (herbarium records OSU and BGSU). However, the Northern Ohio site is now one of the only two locations in the state where *C. candidum* remains (ODNR, pers. comm.). This site has a large, actively managed prairie area (∼900 ha) with wooded areas intertwined. Vegetative cover at this location is dominated by *Andropogon gerardii*, *Sorghastrum nutans* and *Silphium terebinthinaceum*, with *Viola* spp., *Sisyrinchium montanum* and *Fragaria virginiana* co-flowering with *C. candidum*. The prairie is maintained through controlled burns in early spring approximately every 3 years (J. Windus, ODNR, pers. comm.), producing large, thriving populations (total *N* ∼ 6000) in the calcareous soil, although our access to the area was restricted to a subset of the total population by the Ohio Division of Natural Resources.

### 2009 Study

In early May 2009, we established three randomly selected 60-m line transects through a patch of *C. candidum* (*n* > 250). The population density of *C. candidum* at the prairie was previously estimated at 3.26 plants m^−2^ (range = 1–9 plants m^−2^; SD = 2.68) (Walsh 2013). At 5-m intervals, the plant closest to a transect was selected and a second plant of equal size (number of stems and flowers) was chosen within 0.5 m of the transect on the opposite side (total *N* = 72). Any plants with flowers that had already opened, or had any pollinia removed or deposited prior to set up of the experiment were excluded from the study, resulting in the exclusion of one sample point. The standard method of assessing pollen limitation compares the female fitness of open-pollinated plants with that of plants that have had all of their flowers hand pollinated (Wesselingh 2007) to avoid redirection of resources from non-pollinated flowers, which may bias information on fruit or seed set (Knight *et al*. 2006; Wesselingh 2007). One of the paired plants was randomly chosen to receive a hand pollination treatment on all its flowers (mean = 2.06, SE = 0.14, range 1–7) with pollinia from a different population from a site at least 100 m away, while the other member of the pair was open pollinated. The numbers of flowering stems, total stems, leaves per stem and the height of each flowering stem (to the nearest 0.1 cm) were also recorded. Because the site was burned in 2009 ca. 1 month prior to sampling, surrounding vegetation was sparse during the flowering period, precluding the collection of surrounding vegetation data.

Each flower received a single pollinium from a mixed batch of pollinia gathered earlier the same day from a different population at least 100 m away. After hand pollination, flowers were bagged with a mesh (mesh opening size 3 mm × 3 mm) to prevent accidental removal of the pollinium. Mesh bags were removed after all flowers in the experiment had dehisced (ca. 2 weeks) to allow weevil predation. Capsule development was recorded 1 month after floral dehiscence (June), as well as at maturity in August when all capsules were collected and scored for insect damage and seed production. Fruit abortion was scored as the number of fruits initiated in May minus the number of fruits matured in August. We saw no evidence of browsing or herbivory other than weevil damage during the experimental period. Mature capsules were dried at 60 °C for 3 days prior to separate weighing of capsule and seed masses. Mature capsules were scored as predated when circular insect exit holes, ca. 1 mm in diameter, weevil body parts (possibly molts) and a lack of mature seeds were observed.

### 2011 Study

In May 2011 prior to flower opening, we set up a second pollen limitation study on a different nearby population of similar size to the 2009 study on the same property. In this experiment, a hand self-pollination treatment was added, stem and flower number were controlled and fruits were protected from weevil predation to obtain enough fruits for analysis of the effect of pollen quality on fruit set and seed mass. Five 50-m transects were randomly located across the population. For this study, only three-flowered, three-stemmed individuals were chosen to control for plant size, apply the three treatment types and to limit resource reallocation issues that might arise if non-experimental flowers set fruit. At 5-m intervals along each transect, we tagged the nearest three-flowered, three-stemmed individual and wrapped unopened flowers in a mesh to prevent visitation and weevil predation. Each stem was randomly assigned to one of three treatments: hand self-pollinated, hand-outcross pollinated (from a population >100 m away) or open pollination. Plants were checked daily for open flowers and treatments were applied when the stigma became receptive. Open-pollinated stems had mesh bags removed as soon as flowers opened, while hand-outcross and hand-selfed flowers were re-bagged after receiving appropriate treatments. Following floral dehiscence, the pollinator exclusion bags were removed. Initial fruit set was scored 2 weeks after flower dehiscence, with green, enlarged fruits scored as pollinated and pale, shrunken or missing fruits scored as a failed pollination. All fruits at this time were covered in dialysis tubing and secured at both ends to exclude insect damage. Fruit abortion was scored 4 weeks after flower dehiscence as well as at the end of the study (August 2011). We collected fruits 3 months after flower dehiscence, dried them at 65 °C for 48 h and then weighed dissected seed mass to the nearest hundred-thousandth of a gram on a Mettler AE-240 scale (Mettler-Toledo Inc.). We estimated the effect of inbreeding depression (*δ*) on female reproductive success by calculating the mean per-family seed production for each maternal family that matured capsules on both the self and outcross pollination treatments as *δ* = 1 − (*ω*_s_/*ω*_x_) where *ω*_s_ is the seed mass produced by selfing and *ω*_x_ is the seed mass produced by hand-outcross (Johnston and Schoen 1994).

#### Data analysis

Analyses were performed using JMP v.9.0.2 (SAS Institute, Cary, CA, USA, 1989–2013). A generalized linear model (GLM) with an identity link function was used to assess the effects of treatment, average flowering stem height, number of flowers, number of stems and the interaction between treatment and average flowering stem height on percentage of initial capsule development (number of capsules produced/number of flowers), proportion of capsules matured and proportion of capsules preyed upon. The number of flowers and number of stems were not significant variables in any initial models and therefore we used pooled error terms to test for other effects in the final models. The effects of number of flowers, number of stems, average height of flowering stems, treatment and percentage of capsules produced on the probability of abortion, final fruit and seed mass were analysed using a GLM. The proportions of fruit set and predation were arcsin square root transformed and all count data were log transformed. To test for proportionality of increase in response to size variables we used an ln–ln regression, testing for a slope of one (Klinkhamer and de Jong 2005; Karron and Mitchell 2012). For the 2011 study, a GLM with an identity link function examined the effect of pollination treatments on initial fruit set, final fruit set and seed mass, followed by one-way ANOVAs to test for differences among treatment groups on fruit set, abortion rates and final seed mass. *Post hoc* analyses on the one-way ANOVA's were performed using a Tukey–Kramer HSD.

## Results

### 2009 Study

Plants in the study had an average of 2.06 flowers (SE = 0.14, range 1–7) and 3.05 stems (SE = 0.23, range 1–9) per individual. Mature flowering stems had an average height of 22.6 cm (SE = 0.47), ranging between 15.1 and 40.9 cm. The average number of leaves on a plant varied little, with an average of 3.2 leaves per stem (SE = 0.01, range 3–4). Leaf length and width were not measured in this study based on prior work (Walsh 2008) that showed little between-individual variation.

**Figure 1.**
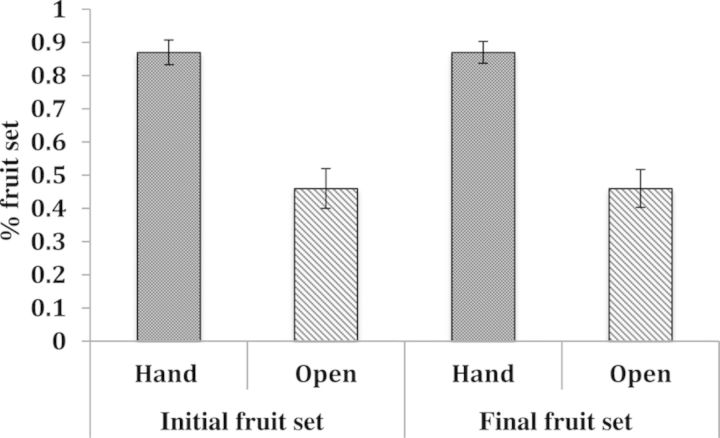
Effect of hand and open pollination treatments on the mean per cent of initial and final fruit set. Plants receiving supplemental pollen produced higher fruit set for both the initial time period (Hand mean = 0.87, SE = 0.037; Open mean = 0.46, SE = 0.06; *t* = −4.11, *n* = 36, *P* < 0.001) and final time period (Hand mean = 0.87, SE = 0.033; Open mean = 0.46, SE = 0.057, *t* = −4.30, *n* = 36, *P* < 0.001).

In these experiments, *C. candidum* showed strong pollen limitation. Initial fruit set, measured 1 month after floral dehiscence, significantly increased in plants receiving supplemental pollen compared with open-pollinated flowers (pollination treatment *P* < 0.0001; whole model *P* < 0.0001, df = 5, AIC = 73.6, *R*^2^ = 0.33; Fig. 1). Plants that received supplemental pollen had an initial fruit set of 87 % (SE = 0.037), while plants only serviced by pollinators had substantially lower initial fruit set (mean = 46 %, SE = 0.06, Fig. 1). Number of flowers and number of stems did not significantly predict the percentage of initial or final capsules produced and were omitted from further analysis. In the subsequent GLM analysis (whole model: *P* < 0.0001; df = 3; AIC = 71.2; *R*^2^ = 0.29), initial fruit set was similarly affected by pollination treatment (*P* < 0.0001), but was significantly influenced by flowering stem height only for open-pollinated plants (interaction effect of treatment × the average flowering stem height, *P* = 0.02; main overall effect of height *P* = 0.11).

**Figure 2.**
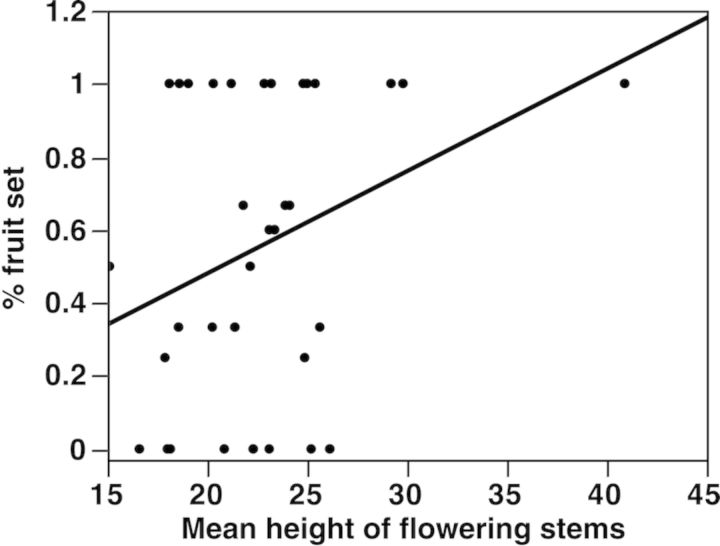
Relationship between per cent fruit set (number of fruits/number of flowers) of open-pollinated plants and the mean height of flowering stems. Linear regression, % fruit set = −0.34 + 0.037 × avg. height flowering stems, *F*_1,35_ = 7.0, *P* = 0.012, *R*^2^ = 0.17.

Final fruit set, measured 3 months post-floral dehiscence, also differed between pollination treatments (model *P* = 0.0004; df = 1; AIC = 121.14; *R*^2^ = 0.16; Fig. 1). Study plants receiving supplemental pollen matured capsules 87 % (SE = 0.87) of the time, while only 46 % (SE = 0.057) of open-pollinated plants fully developed fruit (Fig. 1). However, no measured plant size traits (number of flowers, number of stems, height of flowering stems and number of leaves) or interaction effects explained variation in final fruit set (Fig. 2).

**Figure 3.**
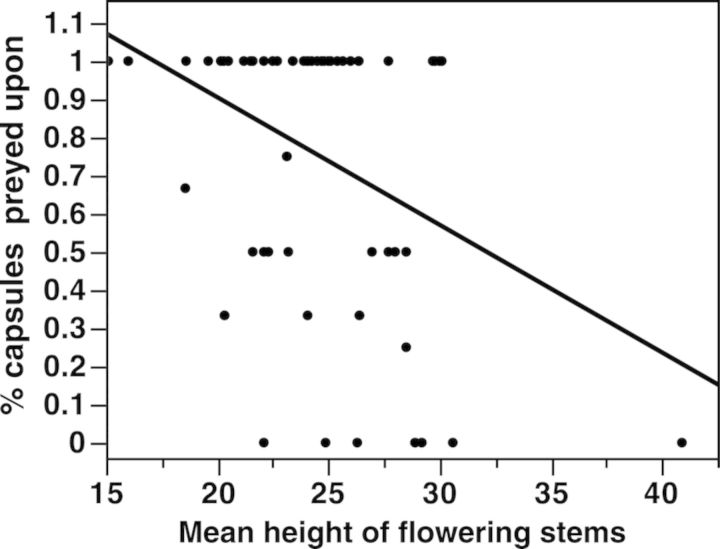
Relationship between the percentage of matured capsules preyed upon (number of capsules preyed upon/number of capsules produced) and the mean height of flowering stems of all plants in the study. Linear regression, % capsules preyed upon = 1.57–0.033 × avg. height flowering stems, *F*_1,61_ = 10.12, *P* = 0.023, *R*^2^ = 0.144.

Of the flowering stems that set fruit, 73 % were preyed upon. Surprisingly, predation rates were not influenced by availability of food for the weevils (numbers of flowers or fruits) or by plant size (number of stems) (whole model *P* = 0.0024; df = 5; AIC = 93.6; *R*^2^ = 0.23). The average height of flowering stems was the only size variable to significantly explain the probability of predation (*P* = 0.002; whole model: *P* = 0.0020; df = 1; AIC = 98.5; *R* = 0.14), with taller stems less likely to be attacked (*F*_1,61_ = 10.1, *P* = 0.002, *R*^2^ = 0.14; Fig. 3). Fruits suffering predation had poor seed production, as the seed mass of predated capsules was 89 % lower than the seed mass of capsules without predation. A total of 22 plants in the study aborted at least one fruit between initial and final fruit set measurements. Seed mass, measured as the dried and extracted seeds from the capsules, increased with increased fruit production (numbers of capsules, *P* = 0.0161) and decreased with the proportion of capsules predated (*P* < 0.0001) (whole model *P* < 0.001; df = 6; AIC = 320.5; *R*^2^ = 0.36).

### 2011 Study

**Figure 4.**
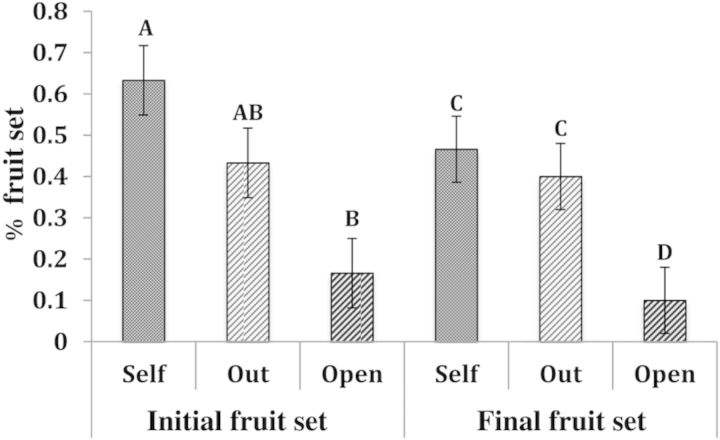
Effect of pollination treatments on initial and final fruit set in the 2011 study. *N* = 30. Mean fruit set for the initial (Self = 0.633, SE = 0.084; Outcross = 0.433, SE = 0.084; Open = 0.166, SE = 0.084) and final fruit maturation times (Self = 0.466, SE = 0.08; Outcross = 0.40, SE = 0.08; Open = 0.1, SE = 0.08) were significantly different as indicated by Tukey–Kramer HSD. Treatments separated by letter indicate significant difference. ANOVA: initial fruit set: *F*_2,89_ = 7.73, *P* < 0.001; final fruit set: *F*_2,89_ = 5.73, *P* = 0.004.

When fruits were protected from weevil predation and plant size and flower number were controlled, initial fruit set varied with pollen source. Plants receiving hand pollination had higher fruit set 2 weeks after floral dehiscence, with plants receiving self-pollen setting 63 % of their capsules (SE = 0.08) and plants receiving outcrossed pollen setting 43 % of their capsules (SE = 0.09). In contrast, fruit set on open-pollinated plants was considerably lower, with only 16 % (SE = 0.06) of open-pollinated flowers initially setting fruit. Initial fruit set differed among pollination treatments (*F*_2,89_ = 7.73, *P* = 0.0008; Fig. 4). Plants that received hand self-pollinations had a significantly higher initial fruit set than the open-pollinated plants (*P* < 0.05, Fig. 4).

**Figure 5.**
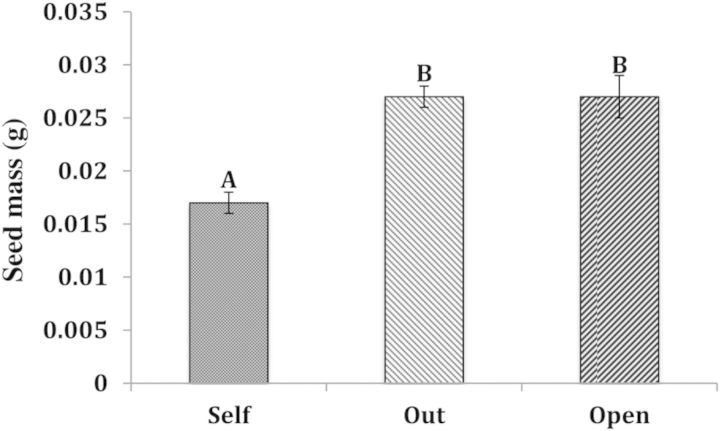
Effect of pollination treatment on seed mass (g) per fruit. The outcross (mean = 0.027, SE = 0.001) and open-pollinated plants (mean = 0.027, SE = 0.002) produced significantly higher seed mass than the self-pollinated plants (mean = 0.017, SE = 0.001) as indicated by Tukey–Kramer HSD, *N* = 29. Treatments separated by a different letter are significantly different. ANOVA: *F*_2,28_ = 18.72, *P* < 0.001, *R*^2^ = 0.59.

Final fruit set, scored 12 weeks after floral dehiscence, was affected by the pollination treatments (*F*_2,89_ = 5.73, *P* = 0.0046; Fig. 4). As in the 2009 study, plants receiving natural pollinator service were pollen limited, as self and outcross hand-pollinations matured more fruits (self mean fruits per flower = 0.46, *P* = 0.0058; outcross = 0.4, *P* = 0.0292) compared with open-pollinated stems (mean fruits per flower = 0.1; SE = 0.081). However, pollen quality did not influence the probability of fruit maturation, as the fruit set of selfed and outcrossed hand-pollination treatments were similar (*P* = 0.83; Fig. 4). However, pollen quality of the pollination treatment did significantly influence final seed mass (*P* < 0.0001, *F*_2,28_ = 18.72, Fig. 5). The open and outcross treatments produced significantly larger seed mass per capsule than the self-pollinated treatments (open = 0.027 g, SE = 0.002; outcross = 0.026 g, SE = 0.001; self = 0.017 g, SE = 0.001), while seed masses of the open pollinated and outcross hand-pollination treatments were similar (Fig. 5). There was no significant difference in abortion rates among treatments (*F*_2,89_ = 1.79, *P* = 0.172). A power analysis indicated that 81 replicates would have been needed to reach a significance level of *P* < 0.05.

When the 12 individuals that matured fruit on both hand-pollination treatments were used to estimate the effect of inbreeding depression on seed production, most individuals showed increased seed mass when pollinated with outcrossed pollen (mean *δ* = 0.463; SD = 0.31). However, the effect of selfing was variable across families. Nine families had an 11–67 % reduction in seed mass when selfed, two families matured a fruit but failed to produce any selfed seed within the fruit, while one family produced similar masses of seed in both the outcross and self hand-pollination treatments.

## Discussion

Orchid species have been routinely shown to demonstrate consistent pollen limitation across multiple years (Snow and Whigham 1989; Ackerman and Montalvo 1990; Calvo 1990; Primack and Hall 1990; Dudash and Fenster 1997). Furthermore, deceptive orchids often produce only half as many fruits as their non-deceptive counterparts (Johnson and Bond 1997; Neiland and Wilcock 1998; Tremblay *et al*. 2004; Jersáková *et al*. 2006). This study provides strong evidence of pollen limitation in a deceptive orchid over two flowering seasons. In 2009, we observed a moderate fruit set from open pollination (46 %), while fruit set was greatly reduced (16.6 %) for *C. candidum* plants in 2011. This number closely parallels open-pollinated fruit set seen across multiple non-burn years at this site (16.5 %; Walsh 2013), as well as levels reported in other relatives, such as 10.5 % in *C. calceolus* (Kull 1998) and 5–13 % in *C. acaule* (O'Connell and Johnston 1998). Hand-pollinated fruit set was consistent between study years and burn/non-burn years, with at least 40 % of flowers setting fruit when supplemental pollen was provided. The mean temperature for the flowering month did not differ between 2009 and 2011 (15.5 °C); however, the 2011 study year received more than double the precipitation (18.5 cm) compared with 2009 (8.8 cm).

Pollen limitation, in principle, has two components, pollen quantity and pollen quality (Aizen and Harder 2007). In plant systems producing normal, dust-like pollen and large numbers of ovules, inadequate saturation of the stigmatic surface may result in only partial pollination. Orchids produce pollen aggregated into sac-like pollinia containing large amounts of pollen, although misplacement of the pollinia on the stigmatic surface by pollinators may result in incomplete pollination. Studies in the deceptive *Dactylorhiza* orchid have shown that flowers may need multiple visits in order to receive enough pollination for complete seed set (Sletvold and Ågren 2010; Sletvold *et al*. 2010). Although Aizen and Harder (2007) argue that pollen supplementation often involves high-quality outcross pollen that could inflate pollen deposition estimates, our 2011 study found that pollen quality manipulation in this system did not significantly increase fruit production, although it did significantly increase seed mass. Different experiments from both years show no significant change in seed mass/fruit between open-pollinated and hand-outcrossed flowers, suggesting that open-pollinated flowers are usually outcrossed.

Although numerous studies have shown that an increase in floral display and plant size increases pollen receipt and fruit maturation (Peakall 1989; Meléndez-Ackerman and Ackerman 2001; Aragón and Ackerman 2004; Mitchell *et al*. 2004; Li *et al*. 2011), we saw no effect of number of flowers, number of stems or number of leaves on pollen receipt or overall fruit maturation. The population studied was relatively dense for an orchid population, 3.26 plants m^−2^ (range = 1–9 plants m^−2^; SD = 2.68), and the large numbers of closely spaced individuals may have limited the ability to detect any effect of floral display size on fruiting success. However, we found strong evidence that greater flowering stem height increases initial fruit set, suggesting that taller plants were more likely to attract pollinators. Others have shown previously that the height and density of surrounding vegetation affects pollination and fruit production in deceptive orchids. Wake (2007) found increased seed set in *C. candidum* when surrounding vegetation was experimentally reduced, while the height of the flowering stem also increased pollination and fruit production in the closely related species *C. acaule* (O'Connell and Johnston 1998). Similarly, Sletvold *et al*. (2013) found strong pollinator-mediated selection for taller plants in the presence of taller vegetation in the deceptive *D. lapponica*. Given the tall grass prairie vegetation in which *C. candidum* occurs, a taller flower would be more visible to pollinators through the vegetation and therefore be more likely to receive pollinator servicing. Compared with other years (measured in a concurrent demographic survey; Walsh 2013), fruit set was unusually high in the 2009 study, which took place immediately after a controlled burn. This would suggest that both increased visibility to pollinators and increased nutrients from the burn may have contributed to this relatively high fruit set. Furthermore, taller flowering stems with increased sun exposure may offer a warmer microenvironment for the small bee pollinators in early spring, as well as greater opportunity for photosynthesis by developing fruits. Future studies on the effects of flowering stem height on seed dispersal in response to local and landscape variation in vegetation density and height may provide additional insights into the functional role of selection on plant traits affecting floral displays.

The presence of a deceptive pollination system may explain why our results with *C. candidum* are contrary to reports in the literature involving non-orchids. While other plants with larger floral displays attract pollinators from greater distances (Sih and Baltus 1987; Hessing 1988), the absence of a reward may discourage further foraging on the same plant, limiting any increase in fitness that would otherwise occur in a large, multi-flowered rewarding plant. Jersáková *et al*. (2006) cite numerous examples of deceptive orchids with reduced geitonogamy, while nectar addition experiments in deceptive orchids have found dramatic increases in self-pollination when reward is added (Johnson *et al*. 2004; Jersáková *et al*. 2006; Walsh 2013). In a deceptive system, a taller stem may increase the probability of pollinator attraction, but the visitor is expected to quickly depart after receiving no compensation for its efforts. Although food deceptive systems may increase pollen limitation compared with rewarding ones, it may be more advantageous to produce fewer, but higher quality fruits than producing additional lower quality (selfed) offspring, as seen in our 2011 data (in which seed mass substantially decreased with hand self-pollination) and several others (e.g. Tremblay *et al*. 2004; Jersáková *et al*. 2006).

These data describe a potential mechanism driving the classical outcrossing hypothesis, which explains the benefits of deceptive orchid pollination via increased outcrossing (Dafni and Ivri 1979; Nilsson 1983; Ackerman 1986; Johnson and Nilsson 1999; Jersáková *et al*. 2006). In their review of published estimates of inbreeding depression in orchids, Sletvold *et al*. (2012) noted that mean inbreeding depression for seed production was 33 %, regardless of mating system. While most individuals in this study produced greater seed mass on average when receiving outcrossed pollen (leading to a mean inbreeding depression of *δ* = 0.46), this outcome was variable across all families, with one individual producing equal seed mass in both self and outcross treatments. The average number of flowers per plant within this population over a 4-year observation period was 1.75, with ∼51 % of flowers setting fruit (Walsh 2013), indicating that although inbreeding depression in *C. candidum* might be overcome by setting an additional fruit, the floral display architecture (single flower/stem) of *C. candidum* makes this highly unlikely. Furthermore, a true estimate of inbreeding depression would require data on the germination and future growth and reproduction of the offspring and is likely to depend on environmental conditions (Cheptou and Donohue 2011; Murren and Dudash 2012). Although seed packets have been previously used to quantify germination in the field in some orchids (Rasmussen and Whigham 1993; Sletvold *et al*. 2012), attempts to germinate *C. candidum* using this method produced no seedlings over a 2-year study period (Walsh 2013).

In this study seed predators preferentially preyed on fruit with shorter flowering stems, exerting a strong concordant selective pressure reinforcing that of the pollinators. In total, seed predation heavily reduced total reproductive output of the population, with 73 % of all capsule-bearing stems attacked by a seed predator. All capsules appeared to be damaged by the same insect, most likely the weevil in the *Stethobaris* genus previously reported to prey upon *Cypripedium* fruit (Light and MacConaill 2011). Weevil predators in this genus are known to feed upon the leaves, flowers and developing capsules of many orchids, destroying most seeds by ovipositing in the maturing capsules. Contrary to our predictions, neither predation rates nor pollination success was related to other size variables such as numbers of flowers, stems and leaves. Our data suggest these weevil predators may prefer to forage on resources closer to the ground, where a lower predation risk may be associated with cover from litter and canopy vegetation, or where less energy is required to climb shorter stems to reach capsules for oviposition. Although our analyses do not indicate any response to food resource abundance such as the number of fruits on a plant, Recart *et al*. (2013) found a significant increase in abundance of another orchid weevil, *Sethobaris polita*, along with increased floral damage and reduced fruit set on a native Puerto Rican orchid, *Bletia patula*, in sites where an invasive orchid *Spathoglottis plicata* co-occurs. Complex plant–pollinator–seed predator interactions have been documented by others (Strauss and Irwin 2004), although instances of conflicting pressures seem to greatly outnumber instances of concurrent pressure.

## Conclusions

Our study quantifies a three-way interaction between plants, pollinators and seed predators in a deceptive orchid system in which mutualists and antagonists are exerting concordant, reinforcing selection on a plant trait, reproductive stem height. Pollinators visited plants with taller stems more often, while another trait often associated with increasing pollinator visitation, floral display size, had no effect on pollination. Furthermore, measures of plant size, such as numbers of stems and leaves that might influence resources available for fruit maturation, did not affect female reproductive success. Seed predators may be attracted to more easily reached resources that are sheltered by surrounding vegetation and less apparent to their invertebrate predators (Marquis 1992; Palo *et al*. 1993). Additionally, the greater seed mass from both outcrossed hand and open pollination events compared with hand self-pollinations suggests that deceit pollination effectively prevents geitonogamy, so that most pollinations in *C. candidum* arise predominately from outcrossing. Although variable in magnitude and based on a small sample, inbreeding depression reduced seed mass by 11–67 % in all but one family, suggesting that conservation of small, at-risk populations that may be vulnerable to decreased pollination opportunities and increased geitonogamy should focus on facilitating outcrossing to increase recruitment.

In addition to the strong directional selective pressure on flowering stem height from both antagonists and mutualists, the height and density of the surrounding heterospecific vegetation matrix likely enhances this selection for taller flowering stems. The evolution of this complex interaction may hold an important lesson in the conservation of *Cypripedium* spp. and other deceptive plants. Management activity that controls surrounding heterospecific vegetation density and height during the flowering period may increase pollination success and fruit maturation, functioning as a cost-effective method to potentially increase rare plant offspring recruitment by modifying the pre-existing natural selective pressures on the biotic interactions of the system. However, while there have been several studies examining the effect of nectar addition on deceptive orchids (e.g. Johnson and Nilsson 1999; Johnson *et al*. 2004; Jersáková and Johnson 2006), there has been no study to date explicitly examining the demographic consequences of the deceptive pollination strategy in any plant. Although further research across multiple years and populations is needed to quantify the relative importance of pollination limitation and seed predation, orchid restoration efforts may benefit from research on whether management can be specifically targeted to ameliorate chronic pollination limitation or seed predation in orchids. As recently argued by Sletvold and Ågren (2014), spatial and temporal variation in selection mediated by the biotic environment strongly affects the extent of pollinator-mediated selection. We propose that the surrounding vegetation context that provides the arena for plant–pollinator–predator interactions, as well as seed dispersal, is an important, relatively understudied component that should be considered for both managers of species of conservation concern as well as biologists seeking greater understanding of the evolution and functional significance of floral traits in these complex interactions.

## Sources of Funding

Our work was funded in part by an undergraduate research grant through the Science, Engineering and Technology Gateway Ohio (SETGO)-NSF program.

## Contributions by the Authors

This research was completed as part of the R.P.W.'s dissertation studying under H.J.M. P.M.A. participated in the 2011 study as part of the SETGO undergraduate research program.

## Conflicts of Interest Statement

None declared.
